# Oral Sepsis as a Cause of Iritis

**Published:** 1905-07-29

**Authors:** 


### ORAL SEPSIS AS A CAUSE OF IRITIS.

Mr. E. Kenneth Campbell [1] suggests that iritis must be added to the somewhat lengthy list of evils which in recent years have been laid to the charge of oral sepsis. He quotes three cases in which, though a history both of rheumatism and syphilis was entirely wanting, there was in each instance marked evidence of oral sepsis (pyorrhœa alveolaris). Signs of absorption were present in a dirty, sallow complexion suggestive of septic anæmia, and in a sour smell in the breath. All the cases recovered after proper attention to the mouth and gums, combined with the use of mydriatics and the administration of tonics. Mr. Campbell considers that about 50 per cent. of the cases of iritis are due to syphilis, but suggests that even in some of these oral sepsis may complicate the situation, and may thus give rise to a mixed infection.

[1] Lancet, July 22, 1905.

